# Spatially resolved single-cell analysis of transcriptomic changes linked with neuropathic pain in human neuromas

**DOI:** 10.1097/j.pain.0000000000003907

**Published:** 2026-01-22

**Authors:** Martina Morchio, Ishwarya Sankaranarayanan, Diana Tavares-Ferreira, Natalie Wong, Simon Atkins, Emanuele Sher, Theodore J. Price, Daniel W. Lambert, Fiona M. Boissonade

**Affiliations:** a Neuroscience Institute and School of Clinical Dentistry, University of Sheffield, Sheffield, United Kingdom. Morchio is now with the Centre for Regenerative Medicine, University of Edinburgh, Edinburgh, United Kingdom; b Department of Neuroscience and Center for Advanced Pain Studies, University of Texas at Dallas, Richardson, TX, United States; c Eli Lilly and Company, Lilly UK Neuroscience Hub, Bracknell, United Kingdom

**Keywords:** Chemokines, Clinical pain experience, Endothelial cells, Inflammation, Neuroma, Neuropathic pain, Single-cell analysis, Spatial transcriptomics, Trigeminal nerve

## Abstract

Injuries to the trigeminal nerve, responsible for sensory innervation to the face, may occur during routine dental procedures, resulting in the formation of a neuroma accompanied by loss of sensation and/or symptoms of pain. To gain insight into the molecular mechanisms underpinning the sensory changes, single nuclei RNA sequencing and spatial transcriptomics were used to profile the transcriptional landscape at single cell resolution of human trigeminal nerves and neuromas. Cellular and transcriptional changes were identified that correlated with the presence of pain, including an expansion of endothelial cells with a proinflammatory phenotype and overexpression of *HLA-A*, *CXCL2*, and *CXCL8*. Interactome analysis highlighted signaling changes linked with the presence of pain. HLA-A protein expression was confirmed in neuromas and positively correlated with symptoms of pain. Our data provide a detailed spatial overview of the cell types that populate human peripheral trigeminal nerves, in health and injury, and their transcriptional profile. The comparison of painful and nonpainful samples highlights several changes in cellular composition, transcriptome and inferred molecular signaling linked specifically with the presence of pain, and also a novel role for HLA-A in neuropathic pain. Our findings represent a valuable resource for pain research, highlighting the role of inflammation, endothelial cell dysfunction, and chemokine signaling in neuropathic pain.

## Abstract 2

Supplemental Digital Content is Available in the Text.

Transcriptomics of rare human neuromas reveals a novel role for endothelial cell dysfunction and HLA-A in neuropathic pain.

## 1. Introduction

Approximately 300,000 peripheral nerve injuries arise each year in Europe.^15^ In addition to loss of motor and sensory function, a proportion of patients have persistent pain for which there is no reliable treatment.^15^ This is associated with sleep disturbances, depression, and various debilitating psychological problems.^81^ Injuries to branches of the trigeminal nerve, most commonly the lingual and inferior alveolar nerves, can occur as a result of routine dental procedures.^49^ Although peripheral nerves regenerate spontaneously, in some patients, the presence of a gap between the proximal and distal ends, bone fragments, scar tissue, and inflammation may prevent functional reinnervation of the target areas, resulting in the formation of a swollen mass termed neuroma.^107^ The frequency of lingual nerve injuries during oral and maxillofacial procedures varies between 0.6% and 2%, resulting in anesthesia, paresthesia, or hyperesthesia of the floor of the mouth, the lingual gingiva, and the anterior part of the tongue, affecting everyday activities such as speaking, eating, and drinking and potentially leading to altered taste.^5,75,78^ In addition, some patients report the presence of neuropathic pain, often described as a burning sensation.^5^ To treat these symptoms, patients can undergo nerve repair surgery where the neuroma is resected and the nerve ends are surgically reconnected, promoting functional recovery of sensation.^5^

Human lingual neuromas represent a unique resource to investigate mechanisms linked with neuropathic pain. The comparison between samples from patients who have incurred the same type of injury, where some, but not all, report symptoms of pain, enables the identification of factors specifically linked with neuropathic pain, which are independent from the pathophysiological changes associated with nerve injury and regeneration. Previous work on human neuromas highlighted changes in the molecular expression of selected targets including ion channels, MAP kinases, and inflammatory mediators.^9,11^ High-throughput bulk transcriptomics has been used to identify overall differences in nontrigeminal neuromas compared to healthy uninjured controls.^83^ However, detailed characterization of the cellular composition and the transcriptional changes linked with the severity of pain at single cell level in human neuromas has never been performed.

In this work, we sought to characterize the cellular and transcriptional composition of healthy and injured trigeminal nerves and identify molecular changes in human trigeminal neuromas linked with the presence of pain. Single nuclei RNA sequencing (snRNA-seq) was used on samples of healthy and injured human trigeminal nerves to characterize the cellular composition at single-cell resolution. Spatial transcriptomics was performed on a larger pool of human lingual neuromas, including both painful and nonpainful samples, to characterize the transcriptional landscape within the morphological context and identify changes in gene expression localized within the nerve fascicles. Cell–cell interactome analysis was performed to identify changes in signaling networks linked with the presence of pain. RNAscope and immunohistochemistry were used to validate the findings.

## 2. Materials and methods

### 2.1. Human lingual neuromas

Lingual neuromas were obtained from patients attending the trigeminal nerve injury clinic at the Charles Clifford Dental Hospital, Sheffield, United Kingdom. Neuromas were collected during nerve repair surgery, performed by Dr. Simon Atkins. All neuromas were collected with the informed consent from the patients, in accordance with ethical approvals received by the NHS Health Research Authority and Sheffield Teaching Hospitals (STH) (19/SC/0308 STH20664). Clinical information including patients' age, sex, and pain history was recorded preoperatively and anonymized.

After surgical removal, the samples were cut longitudinally: one half, intended for snRNA-seq, was immediately flash frozen in liquid nitrogen and stored at −80°C, whereas the other half was fixed for spatial transcriptomics, immunohistochemistry, and RNAscope. Fixation was performed overnight with Zamboni's fixative (0.1 mol/L phosphate buffer, pH 7.4, containing 4% paraformaldehyde and 0.2% picric acid) at 4°C, then the samples were transferred to 30% sucrose overnight at 4°C, placed in optimal cutting temperature compound for rapid freezing on a cryostat freezing plate, and finally stored at −80°C. The tissue was sectioned in a cryostat at a thickness of 10 μm and placed on Superfrost Plus slides (Epredia, 10149870). Sections were dried for 45 minutes and stored at −80°C.

Based on the pain visual analogue scale (VAS) scores, the neuromas chosen for this study were divided in 2 groups: “painful” (pain VAS score higher than 40) and “non-painful” (pain VAS score lower than 15). Differences in VAS scores between painful and nonpainful groups were evaluated using an unpaired *t* test assuming equal variance.

### 2.2. RNA extraction

Quality control of the samples used for spatial transcriptomics was performed with the RNeasy FFPE kit (Qiagen, Hilden, Germany, 73504). The incubation with the deparaffinization solution was omitted because the samples were not embedded in paraffin. The quality of the RNA was assessed using the Bioanalyzer RNA 6000 Pico chip (Agilent, Santa Clara, CA, 5067-1513). Samples with a DV200 higher than 50, indicating that more than 50% of the ribonucleic acids are 200 nucleotides or longer, were deemed to be of satisfactory quality to proceed with the Visium protocol.

### 2.3. Human trigeminal nerve samples

Trigeminal nerve samples were obtained from The Netherlands Brain Bank, Netherlands Institute for Neuroscience, Amsterdam (NBB). All material has been collected from donors for or from whom a written informed consent for a brain autopsy and the use of the material and clinical information for research purposes had been obtained by the NBB.

### 2.4. Nuclei isolation for snRNA-seq

On the day of isolation, utensils including forceps, scissors, and Dounce homogenizers were sterilized and prechilled. Surfaces were cleaned with 70% ethanol followed by RNase Zap. Nuclei isolation media (0.25 M sucrose, 150 mM KCl, 5 mM MgCl_2_, 1 M Tris Buffer pH 8.0) was prepared in advance. Homogenization buffer was made freshly on the day by supplementing the nuclei isolation media with 0.1 mM dithiothreitol, cOmplete protease inhibitor, 0.1% Triton-X, and 0.2 U/μL RNA inhibitor. Wash buffer was made freshly on the day by supplementing the nuclei isolation media with 1% bovine serum albumin and 0.2 U/μL RNA inhibitor. The procedure was performed on ice, using low retention pipette tips and soft touch pipettes to prevent nuclei disruption and maximize recovery.

Briefly, the tissue was chopped up into smaller pieces (<1 mm) using sterile scissors in 2 mL of homogenization buffer on ice. Up to 4 samples were processed in parallel. The homogenate was transferred using a wide bore pipette tip to a glass Dounce homogenizer, further homogenized with a pestle for 15 strokes and left to incubate for up to 2 minutes on ice. The homogenate was filtered through a 70-μm strainer and centrifuged at 800*g* for 7 minutes. The supernatant was discarded, the nuclei were resuspended in wash buffer, centrifuged again, and resuspended in 1 mL 4% formaldehyde fixative solution and fixation buffer (10X Genomics, Pleasanton, CA, PN 2000517). A small aliquot of the sample was used to count the nuclei using trypan blue staining and assess their integrity and the presence of debris and clumping.

### 2.5. snRNA-seq library preparation and sequencing

The samples were prepared for snRNA-seq with the Chromium Single Cell Fixed RNA Profiling for Multiplexed Samples kit (10X Genomics, PN-1000456) according to the manufacturer's instructions. After overnight fixation (16–17 hours), the nuclei from each sample were hybridized with barcoded probes targeting the whole human transcriptome. Two barcodes were used for each sample, targeting to recover 16,000 nuclei per sample. After the Gel Beads-in Emulsion (GEM) generation,^1^ the left-hand and right-hand probes were ligated and the barcoded primers on the gel bead were hybridized to the probes. The probes were extended to include the unique molecular identifier (UMI), the 10x GEM barcode, and a partial TruSeq 1 sequence for Illumina sequencing. Library preparation and sequencing was performed by the Genome Center at the University of Texas at Dallas. Libraries were quality controlled by verifying optimal size using the High Sensitivity DNA Agilent Bioanalyzer kit and sequenced on an Illumina Nextseq 2000.

### 2.6. snRNA-seq data processing and analysis

The reads were demultiplexed, converted to fastq files, aligned to the human genome reference GRCh38, and counted using the Cellranger software provided by 10X Genomics. The h5 files containing the feature barcode matrices were processed with Cellbender to remove ambient RNA.^30^ The parameters used for Cellbender analysis are included in Supplementary Table S1, http://links.lww.com/PAIN/C443. The pipeline was run on a graphics processing unit hosted by the Stanage high performance computing clusters of the University of Sheffield.

Downstream data analysis was performed on R (4.2.3) with Seurat (4.9.9), and the parameters used for data filtering and integration are included in Supplementary Table S1, http://links.lww.com/PAIN/C443. ScDblFinder (1.12) was used to remove doublets from this dataset with default parameters, and barcodes with a doublet score higher than 0.5 were removed from the dataset.^32^ Further filtering was performed removing barcodes with fewer than 500 UMIs, 250 genes, a mitochondrial RNA percentage higher than 5%, and a novelty score (the logarithmic ratio of the number of genes and the number of UMIs detected, indicating the complexity of the RNA species detected) less than 0.8.

The data were normalized with Seurat's SCtransform, with the method “glmGamPoi.” After an initial exploratory analysis of the data, a subset of cell types was identified to be specific to the trigeminal nerve samples, such as a small number of astrocytes, oligodendrocytes, and meningeal fibroblasts, or to the neuromas, such as salivary gland cells or myocytes. To retain the cellular heterogeneity of the different sample types, reciprocal principal component analysis (rPCA) was chosen as the integration method, as it results in fewer overlaps between 2 datasets after integration, enabling the identification of cell types that are unique to each sample type.^60^ The rPCA workflow involved selecting 3000 integration features on the single cell transform (SCT) transformed objects, which are used as anchors to prepare the SCT integration. Principal component analysis is performed on the object split by the sample type, and the integration anchors are identified with the reduction “rPCA” using 30 dimensions. Finally, the data are integrated using the previously identified anchors with 30 dimensions, with the normalization method “SCT.”

The standard Seurat workflow was performed for downstream analysis, involving scaling the data, running the uniform manifold approximation and projection dimensional reduction technique (30 PCs), and finding nearest neighbors (30 PCs) and clusters at resolution 0.5. The clusters were annotated using marker genes obtained from the literature, summarized in Supplementary Table S2, http://links.lww.com/PAIN/C443. Markers for each cluster were calculated with the function FindAllMarkers from Seurat. One cluster was removed as it exhibited high expression of *NEFL, TAC1, CALCA*, and other neuronal genes and was deemed to primarily contain ambient RNA genes deriving from multiplexed samples, which were run on the same chip.

### 2.7. Visium spatial transcriptomics

Spatial transcriptomics was performed with the Visium Spatial Gene Expression for FFPE kit for the Human Transcriptome (10X Genomics, 1000338). Briefly, fixed-frozen sections of human neuromas embedded in optimal cutting temperature compound were sectioned at a thickness of 7 μm, placed on the capture areas of Visium slides, and dried for 45 minutes. Deparaffinization was omitted, and the sections were stained with hematoxylin and eosin (H&E) following manufacturer's instructions. The slides were mounted with 100% glycerol and cover-slipped without sealing. The tissue sections were imaged through the glass slide with a Leica Thunder DMi8 inverted microscope equipped with a FlexaCam C1 Camera system at ×10 magnification. Tile-scans were merged using the Leica Application Suite X (LAS X) software. The coverslip was removed, and the slide was placed in the Visium cassette. Decrosslinking for 1 hour at 70°C with Tris-EDTA buffer was performed to reverse the formaldehyde bonds and expose the nucleic acids. The sections were hybridized overnight with probes targeting the whole human transcriptome: 19,144 genes are targeted by the probe set, and 17,943 genes without off-target activity are filtered and present in the final output. After probe hybridization, stringent washes with 2X saline-sodium citrate buffer were performed to remove nonspecifically bound probes. Then, the left-handed and right-handed probes were ligated with a ligase enzyme, and the RNA was digested to allow the release of the probes, which were captured by the oligos present on the surface of the Visium slide. The probes were extended to incorporate the unique molecular identifier, the spatial barcode information, and the TruSeq Read 1 index. The extended probes were eluted in 0.8M KOH and transferred to PCR tubes. Quantitative PCR was performed on 1 μL of each library with the KAPA SYBR FAST qPCR Master Mix (Merck, Gillingham, United Kingdom, KK4600) to estimate the number of PCR cycles required to amplify each library. Library amplification was performed with a unique 10X Sample Index from the Plate TS Set A (10X Genomics, PN-1000251) for each sample. Short fragments were removed with SPRIselect magnetic beads (Beckman Coulter, Amersham, United Kingdom, B23317). Quality control was performed with the High Sensitivity DNA kit (Agilent, 5067-4626) on a Bioanalyzer. The libraries were sequenced on a NovaSeq 6000 S4 platform with 2 × 150 cycles (PE150) by Novogene Co., Ltd (Cambridge, United Kingdom).

### 2.8. Visium data processing and analysis

Sequencing files in fastq format were processed with Spaceranger (2.1, 10X Genomics) to align the reads to the human reference assembly GRCh38 and process H&E images to align the fiducial frame and detect the tissue. Downstream processing was performed with the R package Giotto (3.3.1): data from each section were merged, genes were filtered so that the expression threshold would be 1, and they were detected in a minimum of 2 spots, whereas spots with less than 100 genes were excluded. Each sample was normalized with a scale factor of 6000. Principal component analysis was calculated using the highly variable features expressed in more than 3% of spots with a minimum average detection threshold of 0.4. The sections were integrated using the Harmony method, uniform manifold approximation and projection and nearest network were calculated to perform Leiden clustering at resolution 0.4 with 1000 iterations. Marker genes for each cluster were calculated with the scran method on the normalized gene expression values.

### 2.9. Differential abundance analysis

Differential cluster abundance was calculated with edgeR (4.2) using the quasi-likelihood negative binomial generalized log-linear model.^3,4^ An EdgeR object was created with the function DGEList, and the design was formulated to include the pain status with a blocking factor as the sample of origin of each replicate. The dispersion was estimated for each cluster with estimateDisp. Differences in abundance were tested with glmQLFTest. The logFC, nominal *P* value, and Benjamini-Hochberg-adjusted *P* value for each cluster are reported in Supplementary Table S3, http://links.lww.com/PAIN/C443.

### 2.10. Gene ontology analysis

Gene ontology (GO) analysis was performed with topGO (2.56) using the significant marker genes (*P* < 0.001) for each cluster. All genes in the spatial transcriptomics dataset were used as background. The GO database was created with biomaRt (2.60), using the “hsapiens_gene_ensembl” as database. Genes were annotated for their biological process and associated gene ontology terms. Gene ontology analysis was run with the topGO function runTest, selecting the “classic” algorithm with “fisher” statistics. *P*-values were adjusted with the Benjamini–Hochberg method. Enrichment is defined as the number of annotated genes observed in the input list divided by the number of annotated genes expected from the background list.

### 2.11. Parametric analysis of gene set enrichment

Cell-type parametric analysis of gene set enrichment (PAGE) was performed using the marker genes from cell types identified with snRNA-seq within the Giotto suite. Parametric analysis of gene set enrichment calculates an enrichment score based on the fold change of cell type marker genes for each spot. Markers for each annotated cluster in the snRNA-seq dataset were identified with the scran method on normalized expression values with Giotto, selecting the top 50 markers with logFC > 1. Parametric analysis of gene set enrichment was calculated with the Giotto function runPAGEEnrich, using a signature matrix that includes the cell types and the top 10 marker genes for each cell type.

### 2.12. Differential expression analysis

The feature count matrices in h5 format were loaded in R with Seurat (4.9.9).^36^ The barcodes overlaying nerve fascicles were manually selected on Loupe browser (6.5, 10X Genomics) and exported in Seurat. When multiple sections were placed on the same capture area, the nerve fascicles from each section were classified as separate technical replicates. The counts from each section were aggregated for pseudo-bulk differential expression analysis, performed with DEseq2 (1.44).^59^ After performing variance-stabilizing transformation with the vst function from DEseq2, PCA analysis was conducted with the package PCAtools (2.16). Differentially expressed (DE) analysis was performed with DEseq2. Nominal *P* values were corrected for multiple testing using the Benjamini–Hochberg method, and the logarithmic fold changes were shrunk with the approximate posterior estimation for generalized linear model (apeglm) method to reduce the variance of logarithmic fold changes caused by low or variable gene counts.^111^ Genes with an adjusted *P*-value lower than 0.05 were deemed to be differentially expressed. The volcano plot and graphs of normalized counts were generated with the R package ggplot. The expression of the top DE genes in the snRNA-seq dataset was visualized with Seurat and ggplot.

### 2.13. Inference of cell–cell communication

CellChat v2 (2.1.2) was used to investigate changes in cell–cell interactions between the clusters in the Visium data in painful and nonpainful samples. CellChat is based on a manually curated database, CellChatDB, which takes into account ligand–receptor (L–R) interactions as well as the presence of cofactors and multimeric complexes. Intercellular communication is calculated based on a mass action model, along with differential expression analysis and statistical tests on cell groups.^47^

The count matrices of all samples, separated by painful and nonpainful, and the cluster annotation from Harmony integration performed with Giotto were used to create the cellchat objects, using the identities of Schwann cells (SC), endothelial cells (Endo), perineurial cells (Peri), B cells (Bcells), and macrophages (Macro). The spatial information from json files generated by spaceranger were used to account for the distances between barcodes. Standard cellchat workflow was used for the generation of the cell–cell interaction inference network, using the human ligand–receptor database. Dysfunctional signaling was identified with differential expression analysis. The interactions were visualized with the plotting functions provided by CellChat, including chord plots, circos plots, and heatmaps.

### 2.14. RNAscope

RNAscope in situ hybridization multiplex version 2 was performed as instructed by Advanced Cell Diagnostics (ACD) following the fixed-frozen tissue preparation protocol.^2^ Antigen retrieval was performed in a steamer at 95°C for 5 minutes with ACD 1X Antigen Retrieval Reagent. Protease treatment was performed with Protease III at room temperature for 1 minute. RNA quality in all tissues was checked by using a positive control probe cocktail (ACD), which contains probes for high, medium, and low expressing mRNAs expressed in all cells (ubiquitin C, peptidyl-prolyl cis-trans isomerase B, and DNA-directed RNA polymerase II subunit RPB1). A negative control probe against the bacterial DapB gene (ACD) was used to reference nonspecific/background label. The following probes were used: RNAscope 3-plex *Hs-SOX10-C1*, targeting *SOX10*, and RNAscope *3-plex Hs-PTGDS-C2*, targeting *PTGDS*.

### 2.15. Immunohistochemistry

Frozen sections of human neuromas cut at a thickness of 10 μm and placed on Superfrost Plus slides were thawed and washed in phosphate-buffered saline (PBS)-T (0.2% Triton-X in PBS). The following primary antibodies were used: mouse monoclonal anti-human leukocyte antigen (HLA)-A (Abcam, Cambridge, United Kingdom, ab52922, 1:1000), mouse monoclonal anti-nerve growth factor receptor (NGFR) (Abcam, ab3125, 1:200), rabbit polyclonal anti-PI16 (Atlas Antibodies, Stockholm, Sweden, HPA043763, 1:200), mouse monoclonal anti-TUJ1 (BioLegend, London, United Kingdom, 801202, 1:100), rabbit polyclonal anti-GLUT-1 (Abcam, Ab15309, 1:400), and mouse monoclonal anti-CD45 (Abcam, Ab8216, 1:400). Labeling of endothelial cells was performed with fluorescein conjugated Ulex Europaeus Agglutinin I (UEA-I) (Vector Laboratories, Newark, CA, FL-1061, 1:50). The sections were incubated with 20% normal donkey serum (NDS) in PBS-T for 1 hour at room temperature. Primary antibodies were diluted in 5% NDS in PBS-T and incubated overnight at 4°C. The slides were washed with PBS-T and incubated with a secondary antibody diluted in 1.5% NDS in PBS-T for 90 minutes in the dark. Sections were mounted in Vectashield Antifade Mounting Medium with DAPI (Vector Laboratories, H-1200-10), cover-slipped, and sealed.

### 2.16. Image acquisition

Images were acquired using a Leica DMi8 inverted microscope fitted with a Leica K5 sCMOS microscope camera system. The microscope was equipped with a LED 3 fluorescent light source in the 390 to 680 range, and 4 filter cubes for epifluorescence excitation: DAPI, FITC, Cy3, and Cy5. Tile scans and z-stacks were acquired at ×20 and ×40 magnification and processed on the Leica Application Suite X (LAS X) software and on Fiji (v2.14).^85^

Images of *PTGDS* and *SOX10* RNAscope were acquired on an Olympus FV3000RS Confocal Laser Scanning inverted microscope at the University of Texas at Dallas. The samples were imaged with 405 nm, 488 nm, 561 nm, and 640 nm diode laser lines at ×40 magnification. Images were acquired on the Fluoview acquisition software and processed on Fiji.

### 2.17. Image analysis and quantification

Image analysis was performed in Fiji (2.14) by a blinded investigator. Maximal intensity projections were generated from z-stacks of tile-scans of the tissue sections imaged at ×20 magnification. A threshold was selected to isolate areas positive for HLA-A labeling, and the percentage of the area within the nerve fascicles, identified by TUJ1 labeling, was recorded. Statistical analysis was performed using GraphPad Prism (10.0.2, GraphPad software, San Diego, CA). Pearson correlation test was used to test the correlation between the area of HLA-A positive labeling with the patient's self-reported pain VAS scores.

### 2.18. Data and code availability

Raw sequencing data will be deposited on the European Genome-Phenome Archive (EGA). Datasets will be released to the public on manuscript publication. Custom R scripts are available at https://github.com/martina-morchio/pain_human_neuromas. To facilitate access to the study data by the research community, we have built a searchable resource at https://martinamorchio.shinyapps.io/neuroma_atlas/.

## 3. Results

### 3.1. snRNA-seq reveals the cellular composition of healthy and injured human trigeminal nerves

**Figure 1.**
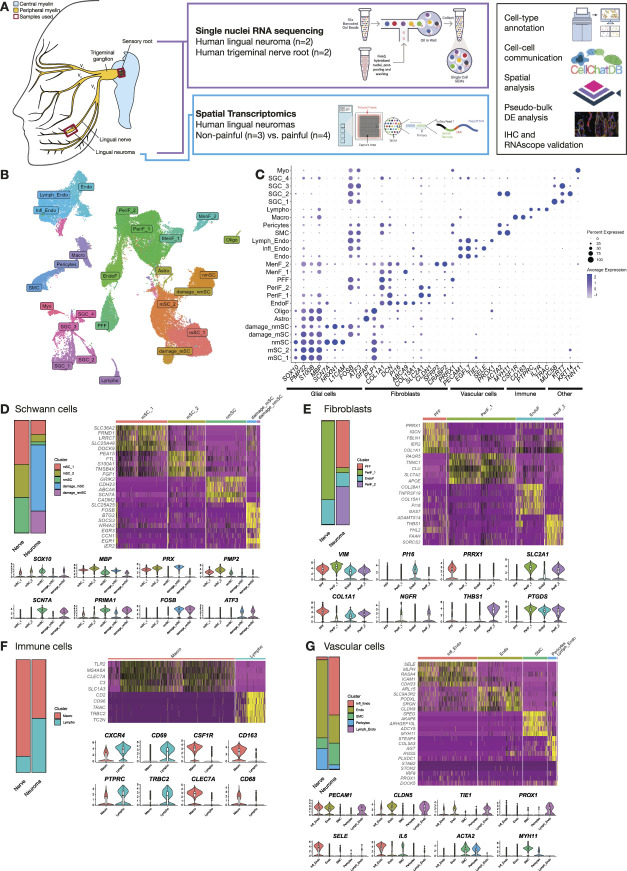
Overview of single nuclei RNA sequencing atlas. (A) Summary of the samples and methods used to characterize the cellular and transcriptional composition of healthy and injured lingual nerves and identify factors associated with neuropathic pain. (B) UMAP of nuclei from human trigeminal nerve roots and lingual neuromas analysed by single nuclei RNA sequencing. (C) Dotplot displaying marker genes used to annotate the cell types in human trigeminal nerves. (D–G) Overview of the major cell types populating trigeminal nerves and neuromas: Schwann cells (D), fibroblasts (E), immune cells (F), and vascular cells (G). For each major cell type, a barplot displays the proportion of each cell subtype in trigeminal nerves and neuromas, a heatmap displays the top marker genes, where yellow indicates a higher level of expression, and violin plots displaying the expression levels of other genes used for annotation. UMAP, uniform manifold approximation and projection.

To characterize the cellular composition of trigeminal nerves with and without injury in humans, snRNA-seq was used on samples of mechanically dissociated trigeminal nerves and neuromas (Fig. 1A). Trigeminal nerves were obtained from the Netherland Brain Bank from 2 donors who died of non-neurological causes and without a diagnosis of dementia (Table 1). Lingual nerve neuromas were obtained from lingual nerve repair surgeries performed at the Royal Hallamshire Hospital in Sheffield, United Kingdom (Table 2).

**Table 1:** Information linked to the samples of trigeminal nerve roots used for single nuclei RNA sequencing.

Sample ID	Clinical code	Tissue	Sex	Age	Collection site
TNR1	2013-022	Trigeminal nerve	F	92	Netherlands Brain Bank
TNR2	2012-104	Trigeminal nerve	M	79	Netherlands Brain Bank

Trigeminal nerve root samples were obtained from the Netherlands Brain Bank from healthy organ donors without diagnosis of other neurological conditions.

**Table 2:** Clinical information and applications of the human neuroma samples.

Sample ID	Time since injury (mo)	Age	Sex	Pain VAS	Spatial	snRNAseq	IHC
LN1	12	27	F	55	Yes		Yes
LN2	7	41	M	2	Yes		
LN3	20	29	F	0			Yes
LN4	19	65	F	100			Yes
LN6	14	25	F	0			Yes
LN7	17	30	F	4	Yes		Yes
LN8	4	36	M	14	Yes		Yes
LN10	69	39	F	0			Yes
LN11	18	33	F	3			Yes
LN12	16	34	F	43	Yes		Yes
LN13	4	28	F	65	Yes	N2	Yes
LN14	13	31	F	0		N1	
LN15	12	34	F	61	Yes		Yes
LN18	17	46	F	72			Yes

Lingual neuromas were obtained during nerve repair surgeries performed at the Royal Hallamshire Hospital in Sheffield, United Kingdom.

IHC, immunohistochemistry; VAS, visual analogue scale.

A total of 86,831 nuclei were sequenced: 54,144 from 2 trigeminal nerve samples and 32,687 from 2 neuroma samples. Ambient RNA was removed with CellBender, and doublets were removed using scDblFinder, removing nuclei with a doublet score higher than 0.5. Nuclei with fewer than 500 reads and 250 genes detected, and a novelty score lower than 0.8 and the presence of mitochondrial genes higher than 5% were removed. After quality control, data from 56,959 nuclei were kept, 21,990 from lingual neuromas and 34,969 from trigeminal root nerves, with a median of 1305 genes detected per nucleus. The data were analysed with Seurat, performing SCT normalization, rPCA integration, clustering at resolution 0.5 to define broad cell types, and a second round of clustering to characterize finer cell types. A total of 25 clusters were identified with annotation markers derived from the literature (Supplementary Table S2, http://links.lww.com/PAIN/C443)^16,17,22,34,58^ (Figs. 1B and C). In the following paragraphs, the clusters identified by snRNA-seq are described in more detail.

### 3.2. Glial cells in human trigeminal nerves and neuromas

The lingual nerve and trigeminal sensory root contain axons of sensory and autonomic neurons, myelinated by Schwann cells or sheathed by nonmyelinating Schwann cells in Remak bundles.^79^ Pan-markers to define Schwann cells include *SOX10* and *MPZ*.^31^ Myelinating Schwann cells additionally express genes linked to myelination such as *MPZ*, *PRX*, and myelination-related transcription factor *EGR2*.^108^ Markers for nonmyelinating Schwann cells include *SCN7A*, *L1CAM*, *NRXN1*, and *NCAM1*.^31,108^ Damaged Schwann cells were marked by the expression of *FOSB*, *JUN*, and *ATF3*.^38,42^ Three clusters were annotated as myelinating Schwann cells: mSC_1, mSC_2, and damage_mSC. mSC_1 and mSC_2 are found in both neuromas and trigeminal nerve root samples and are enriched in markers for myelination such as *PMP22*, *MBP*, and *MPZ* (Fig. 1D). A third myelinating Schwann cell cluster, damage_mSC, was found only in the neuroma samples. Damage_mSC is enriched in markers for myelination (*PRX*) but also genes indicating stress (*AATK*, involved in apoptosis),^104^ inflammation (*TNFRSF25*, member of the TNF family that induces the activation of NF-kB pathway),^90^ and keloid formation (*COL18A1*, involved in ECM deposition and more highly expressed in keloidal Schwann cells compared to healthy skin Schwann cells).^23^ Nonmyelinating Schwann cells (nmSC) were detected in both neuromas and trigeminal nerve roots and display enrichment in genes previously identified as nmSC markers including *NRXN1*, *SCN7A*, and *PRIMA1*.^108^ A damage_nmSC was also identified by the expression of nonmyelinating marker genes such as *SCN7A*, as well as stress and injury markers *FOSB* and *ATF3*, and was only present in the neuroma samples. Damage_nmSC displayed a high expression of genes involved in the injury stress response (*EGR1* and *EGR3*), as well as genes involved in the AP-1 transcription factor complex including *FOSB*, *JUN*, *JUND*, and *JUNB*.

A smaller proportion of the nuclei from the neuromas were classed as Schwann cells (9%) compared to the proportion in nerve samples (45%). Among the Schwann cells in neuroma samples, damage_mSC and damage_nmSC were overrepresented, making up 59% and 20% of all the Schwann cells, respectively (Fig. 1D). This indicates a sharp decline in healthy Schwann cells indicative of demyelination, and the survival and/or proliferation of damage-associated Schwann cells, characterized by the expression of genes involved in the injury stress response.

Yim et al.^108^ identified a myelinating Schwann cell subpopulation in mouse and human nerves that expresses high levels of *PMP2* and preferentially myelinates large diameter axons, in particular motor axons, which might correspond to the mSC_2 cluster identified here, where *PMP2* expression is significantly upregulated. Although motor axons would be found in the motor root of the trigeminal nerve, other large-diameter axons such as Aβ low threshold mechanoreceptors present in the sensory root of the trigeminal nerve might be preferentially myelinated by the mSC_2 cluster.

Central glial cells were identified in the trigeminal nerve roots (Supplementary Fig. S1A, http://links.lww.com/PAIN/C443), where one cluster characterized by *GFAP* expression was annotated as astrocytes (Astro) and one positive with *MOG* and *OLIG1* as oligodendrocytes (Oligo). These clusters were absent in the neuromas and are specifically abundant in one trigeminal nerve root sample, possibly because of the inclusion of the peripheral to central transition zone where peripheral myelin is replaced by central myelin.^71^

### 3.3. Fibroblast heterogeneity in human trigeminal nerves

Fibroblasts are essential for the production of connective tissue to create the structural architecture that supports nerves. Fibroblasts are generally marked by fibrillar collagen genes such as *COL1A1, COL1A2, COL5A1,* as well as collagen-related gene expression such as *DCN*.^67,97^ Fibroblasts are a highly heterogeneous cell type with different functions and gene expression depending on the tissue of origin. In peripheral nerves, fibroblasts' function and phenotype differ based on the position within the fascicular organization of the nerve, characterized by 3 main subtypes: endoneurial, expressing *CSPG2*, *P4HB*, and *CD34*^77^; perineurial, characterized by GLUT-1 protein expression^72^; and epineurial, marked in animal studies by *Sfrp2* and *Pcolce2*.^17,58^ In addition, nerve roots are ensheathed by the meninges, which contain meningeal fibroblasts (MenF), marked by *CRABP2* and *OGN*.^22^

**Figure 2.**
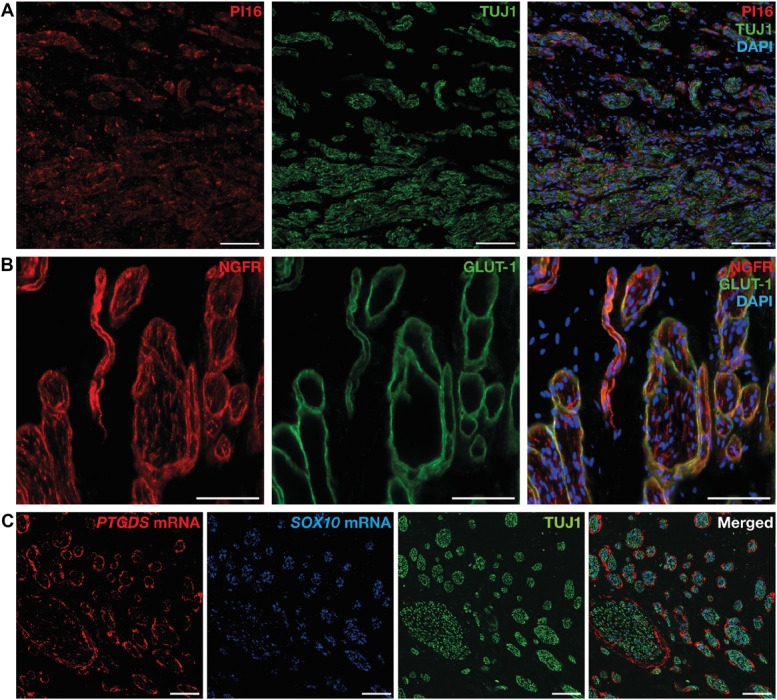
Protein and RNA expression of fibroblast marker genes in human neuromas. (A) Representative image of PI16 immunolabeling in endoneurial-like cells within nerve fascicles, labeled by TUJ1. (B) Representative image of dual-labeling of the p75 NGF receptor (NGFR) and GLUT-1, a marker for perineurial cells, showing colocalization. (C) Representative image of *PTGDS* and *SOX10* RNAscope combined with TUJ1 immunolabeling, highlighting the location of *PTGDS* mRNA in perineurial-like structures surrounding nerve fascicles. All scale bars are 100 µm. Images taken at ×40 magnification. NGF, nerve growth factor.

In our dataset, endoneurial (EndoF), perineurial (PeriF_1 and 2), and profibrotic (PFF) fibroblasts were detected in both sample types (Fig. 1E), whereas in the trigeminal sensory roots, meningeal fibroblasts were also identified (MenF_1-2) (Supplementary Fig. S1A, http://links.lww.com/PAIN/C443). All clusters robustly express the general fibroblast marker vimentin (*VIM*) as well as collagen genes *COL1A1* and *COL1A2* (Fig. 1E). The endoneurial cluster, found in both neuroma and healthy nerve samples, was enriched in *PI16*, *ABCA6, 9* and *10,* and *APOD*. PI16 was identified by Singhmar et al.^87^ to promote pain-like behavior after sciatic nerve injury in rats by inducing permeability of the blood–nerve barrier and increasing immune cell infiltration; however, in the rat sciatic nerve PI16 expression is localized to the perineurial and epineurial layer.^87^ To verify the accuracy of our analysis, we decided to confirm where PI16 expression is localized in the human neuromas by immunohistochemistry. Indeed, we found that PI16 was localized to endoneurial-like cells in human neuromas (Fig. 2A).

PeriF_1 was more abundant in the trigeminal root samples, whereas PeriF_2 was found primarily in the neuroma samples. PeriF_1 was enriched in genes such as *IGFBP6* and *SLC2A1*, widely used as a perineurial marker gene.^72^ PeriF_2 displayed increased expression of *THBS1*, *CCN1*, and *ADAMTS14*, involved in extracellular matrix formation.^53,68,80^ THBS1 is secreted by fibroblasts in peripheral nerves after transection injury, promoting neurite outgrowth.^37^ CCN1 promotes SC proliferation and upregulation of c-Jun.^18^ This suggests increased fibrosis driven by perineurial fibroblasts after injury, potentially interfering with axonogenesis and preventing axons from reaching their targets.

*NGFR* was expressed in the perineurial clusters to a higher level compared with that found in Schwann cells. This was unexpected because NGFR is a well-known marker for repair-Schwann cells^45^; however, there are also reports highlighting NGFR expression in rat perineurial cells.^14,106^ We performed immunohistochemistry to validate whether NGFR was indeed localized to perineurial cells, labeled by GLUT-1, and found that the majority of the signal was localized to perineurial cells, with minor labeling within the nerve fascicles (Fig. 2B). *PTGDS*, encoding for prostaglandin D2 synthase (PTGDS), was also highly expressed in perineurial fibroblasts. Because of its relevance to pain sensitization, we confirmed its expression by RNAscope analysis to perineurial structures surrounding TUJ1+ fibers and *SOX10*+ Schwann cells (Fig. 2C). PTGDS is an enzyme involved in the conversion of PGH_2_ to PGD_2_, leading to neuronal sensitization and pronociceptive effects in both animal models and humans.^44,69,94^

Finally, profibrotic fibroblasts (PFF) were particularly abundant in the neuromas, displaying high expression of the profibrotic marker *PRRX1*.^55^ This cluster was enriched in ECM-related genes including *COL1A1*, *FBLN1*, *COL3A1*, and *COL1A2*, as well as inflammation-related genes such as *FOSB*, involved in promoting a profibrotic program in pulmonary fibrosis^19^ and *LSP1*, which mediates neutrophil activation.^54^

### 3.4. Immune cell populations in trigeminal nerves and neuromas

Two immune cell subpopulations were identified: lymphocytes (602 nuclei) and macrophages (2204 nuclei) (Fig. 1F). The macrophage cluster expressed high levels of *RGS1*, marker for both macrophages and T cells,^7,29^ *CSF1R* and *CLEC7A*, commonly used as specific macrophage markers,^58,102^ and *CD163*, a marker for M2 macrophages.^41^

The lymphocyte cluster displayed high levels of *CXCR4* and *CD69*, T-cell markers,^66^ *CYTIP*, also found to be expressed in T cells,^74^ and *TRBC2*, involved in antigen binding activity.^61^ In the neuroma samples, a higher proportion of lymphocytes are detected compared to macrophages. T cells have been identified to have an important role in nerve repair, modulating remyelination and inflammation.^92^

Finally, 4 subtypes of salivary gland cells (SGC_1, SGC_2, SGC_3, SGC_4) and one cluster of myocytes (Myo) were identified specifically in the neuroma samples (Supplementary Fig. S1B, http://links.lww.com/PAIN/C443).

### 3.5. Vascular cell expansion in human neuromas

Endothelial cells, vascular smooth muscle cells, and pericytes make up the vasculature that supplies peripheral nerves. A total of 9113 nuclei were annotated as vascular: endothelial cells (Endo) were marked by the expression of *PECAM1* and *EGFL7*,^17^ lymphatic endothelial cells by *PROX1*, inflamed endothelial cells (Infl_Endo) by *SELE*, smooth muscle cells (SMC) by the expression of both *ACTA2* and *MYH11*, and finally pericytes, which were positive for *ACTA2* but negative for *MYH11* (Fig. 1G). In the neuromas, 17% of the total nuclei belong to the Infl_Endo cluster, which is almost absent in the nerves, where the majority of endothelial cells were annotated as Endo. Infl_Endo was enriched with genes usually found in cytokine activated endothelial cells and involved in leukocyte recruitment at the site of injury, such as *SELE*,^105^ *ICAM-1*, considered a master regulator of inflammation and injury resolution,^12^ and interleukin-6 (*IL6*), a hallmark of inflammation arising after nerve injury.^82^ This cluster also displays downregulation of tight junction genes such as *CLDN5*, which is linked to the breakdown of the blood–brain barrier.^98^ Overall this shows a marked expansion of endothelial cells in the neuromas with a distinct proinflammatory phenotype.

### 3.6. Spatial transcriptomics identifies distribution of cell types within the morphological context of human neuromas

**Figure 3.**
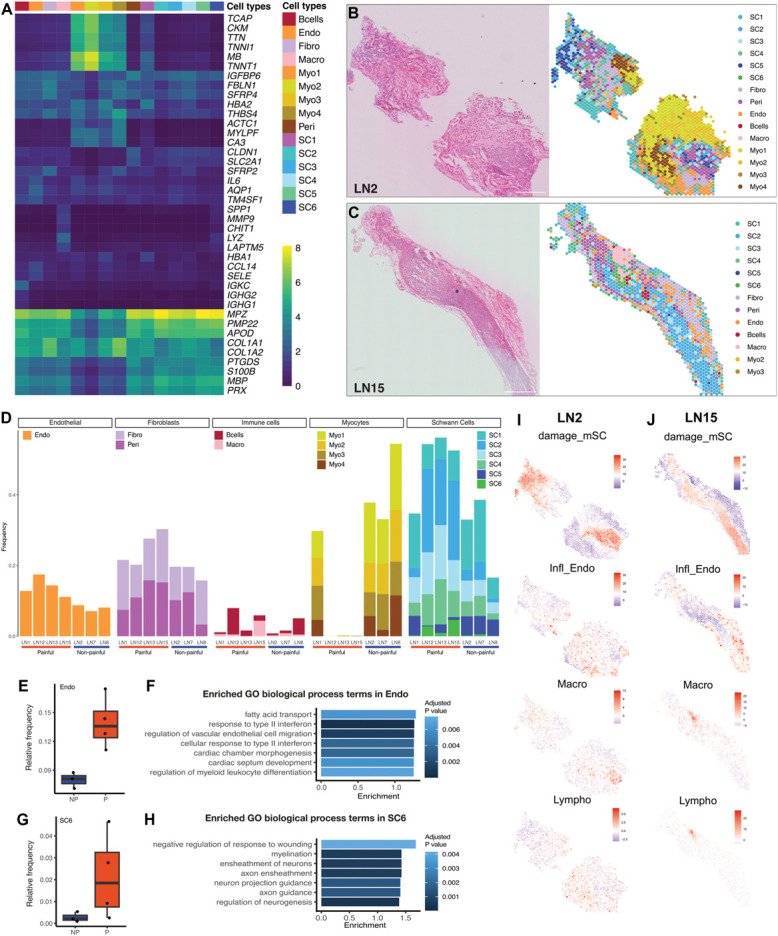
Spatial transcriptomics of human neuromas. (A) Heatmap displaying the expression of marker genes for each cell type identified by Visium analysis in human lingual neuromas where yellow indicates higher expression. (B and C) Representative sections of LN2 (pain VAS: 2) and LN15 (pain VAS: 61) stained with H&E (right) and corresponding spots color-coded by the enriched cell type identified by clustering. (D) Enriched cell-type proportion across samples, divided by major cell type: from left to right, endothelial cells (Endo), fibroblasts (Fibro and Peri), immune cells (Bcells and Macro), myocytes (Myo1–4), and Schwann cells (SC1–6). (E and G) Boxplot displaying the frequency of endothelial cells (E) and one subtype of Schwann cells (SC6, G) in painful and nonpainful samples. (F and H) Enriched GO terms for biological processes in differentially expressed genes in the Endo (F) and SC6 (H) cluster, where the x-axis indicates the enrichment, calculated by dividing the number of significant genes over the expected number of genes in each GO term. (I and J) Representative images of PAGE analysis, where marker genes of the clusters annotated from the snRNA-seq analysis of human neuromas were used to identify the enrichment of specific cell types in the spatial data. Relative enrichment of cell-type gene signatures is displayed on a scale from lowest (purple) to highest (red), where each image has a separate scale bar. The enrichment of gene signatures characteristic of damaged Schwann cells (damage_mSC), inflamed endothelial cells (Infl_Endo), macrophages, and lymphocytes is shown for LN2 (I) and LN15 (J). GO, gene ontology; PAGE, parametric analysis of gene set enrichment; VAS, visual analogue scale.

Visium spatial transcriptomics was used to characterize the spatial distribution of cell types and the transcriptional signature in the morphological context of the tissue. Consecutive sections from 7 human neuroma samples (Table 2) were placed on Visium slides and processed for spatial transcriptomics (Fig. 1A). The data were analysed with SpaceRanger and Giotto, using Harmony to integrate the data and Leiden clustering to identify subpopulations. A total of 17 clusters were identified and annotated based on the genes identified from snRNA-seq and the literature.^16,17,22,34,58^ Spots enriched for fibroblasts, endothelial cells, Schwann cells, perineurial cells, myocytes, B cells, and macrophages were annotated based on the top differentially expressed genes (Supplementary Table S4, http://links.lww.com/PAIN/C443). The heatmap in Figure 3A displays the top marker genes for each cluster, whereas representative sections from a painful sample (LN15) and a nonpainful one (LN2) are shown in Figures 3B and C.

The clusters were differentially distributed across samples (Fig. 3D). Schwann cell clusters (SC1–6), characterized by the expression of myelination genes such as *PMP22*, *MBP*, *PRX*, and *S100B*, were the most abundant in LN12, LN13, and LN15. SC1 displays the expression of both Schwann cell marker genes and hemoglobin genes (*HBA1, HBA2*). Because of its localization to the outer edge of tissue sections, the expression of hemoglobin transcripts might indicate the presence of residual red blood cells after surgical removal of the sample. The other 5 clusters were found across all samples mostly overlaying areas containing nerve fascicles.

The most ubiquitous cluster expressed genes enriched in fibroblasts including *COL1A1*, *FBLN1*, and *COL1A2*.^67^ The cluster was localized to areas surrounding the nerve fascicles, suggesting the presence of fibrotic tissue. This cluster displayed overexpression of *SFRP2* and *4*, encoding for secreted frizzled-related proteins that act as extracellular regulators of the Wnt signaling pathway by competing for Wnt ligand binding.^103^ The Wnt pathway has been identified as a critical mediator of fibrosis, contributing to fibroblast activation and differentiation into myofibroblasts.^13^ Changes in Wnt gene expression have been reported in both human and animal models of peripheral nerve injury, including *SFRP4* overexpression.^96^

The second most ubiquitous cluster (Endo) expressed *AQP1*, *TM4SF1*, and *SELE*, indicating the presence of endothelial cells.^17,40,100^ *IL6* and *CCL14* were also enriched in this cluster suggesting a proinflammatory phenotype of the endothelial cells present in these samples: the role of IL-6 as a proinflammatory molecule is well-known,^91^ whereas CCL14 has been shown to bind to CCR1, 3, and 5, potentially inducing leukocyte infiltration.^35^

The perineurial cluster (Peri) was enriched in *PTGDS*, *CLDN1*, and *SLC2A1*^17,72^ and found across all samples in areas associated with nerve fascicles. In samples LN15 and LN12, which display a more ordered fascicular organization compared to the other samples, this cluster is distributed along the edges of the nerve fascicles, in line with the expected morphology of the perineurial barrier.

Four clusters (Myo1–4) enriched in myocyte markers, such as *MB*, *TNNT1*, *CKM*, and *ACTC1*, were particularly abundant in LN2 and LN8. These clusters overlay areas with a morphology typical of skeletal muscles, characterized by the presence of multinucleated myocytes, and are not directly associated with nerve fascicles.

Finally, 2 clusters were enriched in immune cell markers. The cluster annotated as B cells (Bcells) was enriched in *IGKC*, encoding the Immunoglobulin Kappa Constant, and *IGHG1*–*2*, encoding the immunoglobulin heavy constant gamma 1 and 2, respectively.^39^ This cluster was found across all samples but was particularly abundant in LN12 and LN8. The cluster annotated as Macro was enriched in genes such as *LYZ*, a marker for macrophage activation,^50^ *SPP1*, encoding a protein secreted by activated macrophages,^26^ *LAPTM5*, associated with proinflammatory activation of macrophages,^33^ and *CHIT1*, involved in macrophage polarization and activation.^88^ The macrophage cluster also expressed high levels of *MMP9*, known to be secreted by Schwann cells in the early stages after nerve injury, promoting blood–nerve barrier breakdown and leukocyte influx at the site of injury.^46^ The macrophage cluster was particularly abundant in LN15 (Fig. 3C), one of the samples classified as painful based on the patient's reported VAS score (Table 2).

The location of specific cell types was confirmed using cell-type deconvolution with PAGE.^21^ Examples are shown in Figures 3D–H where the enrichment of damage_mSC, Infl_Endo, macrophage, and lymphocyte gene expression signatures, obtained from the snRNA-seq dataset, is shown across the barcodes of a representative section of LN2 (Fig. 3I) and LN15 (Fig. 3J).

Differential abundance of cell types between painful and nonpainful samples was tested with edgeR^3,4^ (Supplementary Table S3, http://links.lww.com/PAIN/C443). Among the top differentially abundant clusters were Endo (Fig. 3E) and SC6 (Fig. 3G), both more abundant in the painful samples. The endothelial cell cluster was enriched for GO biological processes including response to interferon II and regulation of vascular endothelial cell migration and myeloid leukocyte differentiation (Fig. 3F). The Schwann cell cluster SC6 was enriched for GO terms including negative regulation of response to wounding, axon guidance, and regulation of neurogenesis (Fig. 3H).

### 3.7. Differential expression analysis identifies chemokine signaling and antigen presentation to be linked with neuropathic pain

**Figure 4.**
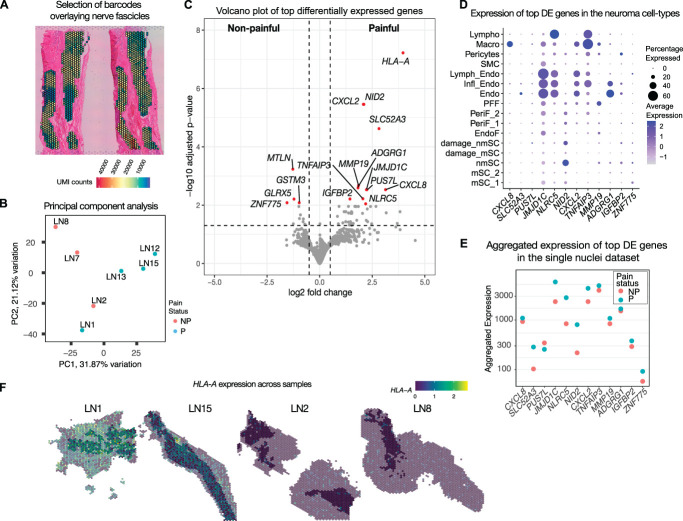
Pseudo-bulk differential expression analysis in painful and nonpainful neuromas. (A) Representative image displaying the selection of barcodes overlaying nerve fascicles in a sample of human neuroma. (B) Principal component analysis (PCA) displaying the distribution of the samples used for pseudo-bulk DE analysis according to the first 2 principal components displayed on the x and y axes. (C) Volcano plot displaying the top DE genes in painful and nonpainful neuromas, where the x-axis represents the logarithmic fold change in expression, and the y-axis represents the adjusted *P*-value. (D) Dotplot displaying the expression of the top DE genes in the cell types identified by snRNA-seq of human neuromas. (E) Plot representing the aggregated level of expression of top DE genes in the painful and nonpainful samples analysed by snRNA-seq in human neuromas. (F) Representative images of the expression of *HLA-A* across 4 samples of human neuromas classified as painful (LN1 and LN15) and nonpainful (LN2 and LN8), where the barcodes overlaying nerve fascicles used for DE analysis are shown in full color, whereas the ones not in the fascicles are opaque, and all barcodes are color-coded to indicate the expression level from low (blue) to high (yellow). DE, differentially expressed.

Pseudo-bulk differential expression analysis was performed by selecting the barcodes overlaying nerve fascicles based on H&E staining, to focus on pathologically relevant areas that are more likely to contribute to the development and maintenance of neuropathic pain (Fig. 4A). Principal component analysis (Fig. 4B) displayed the separation between painful and nonpainful samples. The analysis identified 59 DE genes (*P*adj < 0.05) (Fig. 4C). Among the top DE genes (*P*adj < 0.01) are *HLA-A*, a major histocompatibility complex (MHC) class I gene involved in antigen presentation, chemokine genes *CXCL2* and *CXCL8*, the gene *NID2* encoding for a basement membrane protein, *SLC52A3*, encoding for a riboflavin transporter, the metalloprotease-encoding gene *MMP19,* and *ADGRG1*, encoding for a G-protein–coupled receptor (Fig. 4C). The log-normalized counts are displayed in Supplementary Figure S2, http://links.lww.com/PAIN/C443.

The expression of the top DE genes was confirmed in the single nuclei dataset for the neuromas (Fig. 4D), with the exception of *HLA-A*, as the probes for this gene were absent from the probe set used for snRNA-seq. Several of the DE genes were expressed in endothelial cells, particularly *NLRC5*, a trans-activator of *HLA-A*, *JMJD1C*, a histone demethylase, the chemokine *CXCL2, SLC25A3, ADGRG1*, and *TNFAIP3*, encoding for a zinc finger protein and involved in TNF-mediated apoptosis, also expressed in macrophages and lymphocytes. *CXCL8* was expressed in macrophages, whereas *NID2* was expressed in nonmyelinating Schwann cells. By comparing the expression of the DE genes in the samples used for snRNA-seq analysis, which included one painful and one nonpainful neuroma, the direction of the change in expression was confirmed in all genes except for *PUS7L*, a pseudourinidase synthase, which was upregulated in the painful samples as measured by spatial transcriptomics but displayed lower expression in the painful sample compared to the nonpainful one in the snRNA-seq dataset (Fig. 4E). The expression profile of *HLA-A* in representative sections of 2 painful and 2 nonpainful samples is shown in Figure 4F.

### 3.8. Changes in cell–cell interactions in painful neuromas

**Figure 5.**
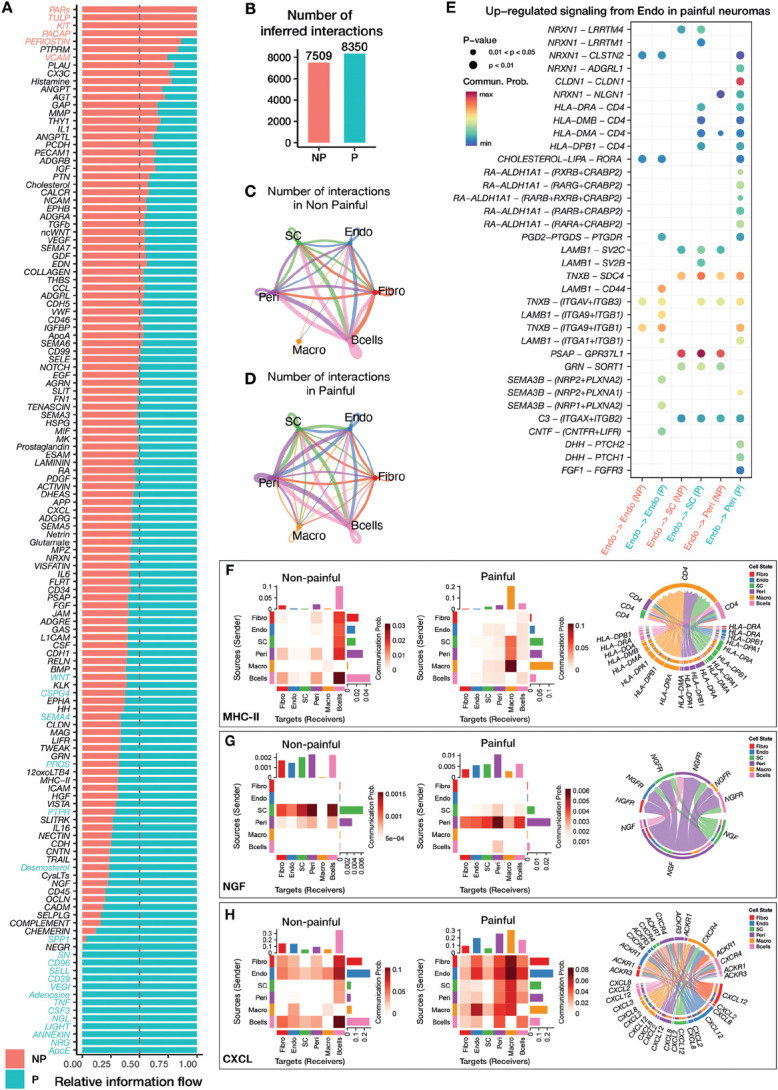
Inferred cell–cell interactions in painful and nonpainful neuromas. (A) Barplot displaying the relative information flow of pathways with significant inferred interactions in painful and nonpainful neuromas. (B) Barplot displaying the number of interactions inferred in painful and nonpainful neuromas. (C and D) Circle plots displaying the interactions between cell types in nonpainful (C) and painful (D) neuromas, where the thickness of the lines is proportional to the number of interactions with a cell type. (E) Bubble plot displaying interactions between ligand–receptor pairs that are differentially expressed in painful and nonpainful neuromas involving endothelial cells, where the size and color of each circle represents the *P*-value and the likelihood of the interaction, respectively. (F, G, H) Heatmaps showing the communication between cell types in painful and nonpainful neuromas in the MHC-II (F), NGF (G), and CXCL (H) pathways, where a darker shade of red represents a higher communication probability, as displayed in the scale (note that the scale is different for each heatmap). The chord plots represent the ligand–receptor pairs exhibiting significant interactions in the painful neuromas. CXCL, C-X-C motif ligand; MHC, major histocompatibility complex; NGF, nerve growth factor.

To begin to understand the cell–cell interactions occurring in painful and non–painful neuromas, CellChat v2 was used on the combined spatial datasets grouped by pain status. CellChat v2 takes into account both the level of expression and spatial location of each ligand–receptor pair from a manually curated database.^48^ The spots were subset and merged to include Schwann cells (SC), endothelial cells (Endo), fibroblasts (Fibro), perineurial cells (Peri), B cells (Bcells), and macrophages (Macro). The postulated differences in information flow between painful and nonpainful samples in pathways with significant interactions are shown in Figure 5A. In total, 8350 interactions were inferred in painful samples, whereas 7509 interactions were inferred in nonpainful ones (Fig. 5B). The change in cell-type contribution of signaling is shown in the chord plot in Figures 5C and D, where line thickness indicates the number of interactions. Overall, the painful samples display increased connectivity among the macrophages, perineurial cells, Schwann cells, and endothelial cells. Analysis of differentially expressed ligands and receptors identified several ligand–receptor interactions upregulated in the painful samples, particularly involving endothelial cells (Fig. 5E), which display an increased likelihood to interact with perineurial cells through CLDN1, DHH, and FGF1 signaling, with Schwann cells through MHC class II molecules and with endothelial cells through the activation of the LIF receptor.

By examining individual pathways, changes in the contribution of cell types can be identified: MHC-II signaling is increased in painful samples (Fig. 5F) where MHC class II ligands are more likely to interact with CD4 expressed within the macrophage cluster rather than in the B cells cluster (Supplementary Fig. S3A, http://links.lww.com/PAIN/C443); NGF signaling is also increased in painful samples where the main source is the perineural cluster, rather than Schwann cells (as for the nonpainful neuromas) (Fig. 5G, Supplementary Fig. S3B, http://links.lww.com/PAIN/C443), and CXCL signaling displays an increased expression of ligands and receptors in perineurial, macrophage, and endothelial clusters (Fig. 5H, Supplementary Fig. S3C, http://links.lww.com/PAIN/C443).

### 3.9. HLA-A protein is expressed in human neuromas and expression in nerve fascicles correlates with symptoms of pain

**Figure 6.**
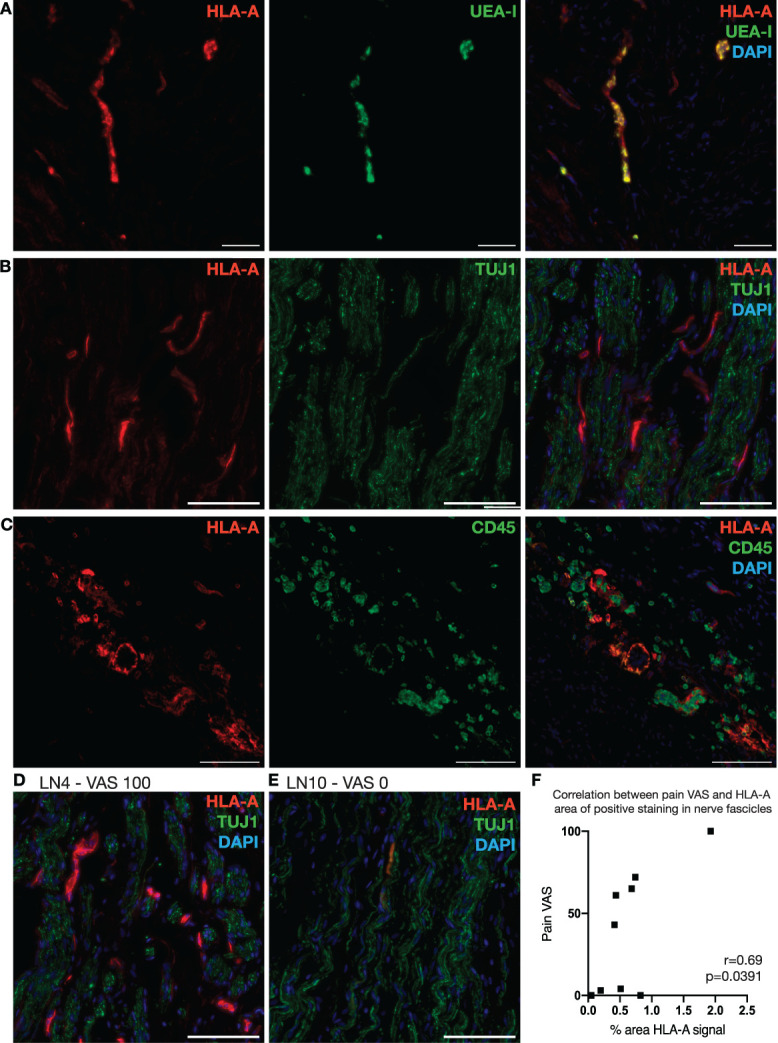
HLA-A immunohistochemistry in human neuromas. (A–C) Representative sections of HLA-A (Cy3) dual-labeling with the endothelial cell marker UEA-I (FITC, A), the axonal marker TUJ1 (FITC, B), and the immune cell marker CD45 (FITC, C) in samples of human neuromas. Scale bar: 100 μm. (D and E) Representative sections of HLA-A (Cy3) signal within nerve fascicles, marked by TUJ1 labeling (FITC) in a painful (LN4, D) and nonpainful (LN10, E) sample of human neuroma. Scale bar: 100 μm. (F) Correlation between the area of HLA-A positive staining associated with nerve fascicles (identified with TUJ1 labeling) and the intensity of pain reported by the patient. Pearson r = 0.69, *P* = 0.0391. FITC, fluorescein isothiocyanate; HLA, human leukocyte antigen; UEA, Ulex europeus agglutinin.

Because of the marked overexpression of *HLA-A* in painful vs nonpainful nerve fascicles, detected with spatial transcriptomics, HLA-A protein expression was further investigated by immunohistochemistry. HLA-A strongly colocalized with UEA-I (Vector Laboratories, FL-1061), a leptin that binds to glycoproteins expressed on the surface of endothelial cells (Fig. 6A). HLA-A positive staining was closely associated with nerve fascicles marked by TUJ1 (Fig. 6B), as well as CD45^+^ immune cells, with some cells displaying colocalization (Fig. 6C). The expression of HLA-A was investigated in a total of 9 samples, as indicated in Table 2, including 4 nonpainful and 5 painful ones. Patients in the nonpainful group reported an average pain VAS score of 1.75, whereas patients in the painful group had VAS scores ranging between 43 and 100, with an average of 68.2. There was a significant difference in the pain VAS score between the high and low pain groups (*P* < 0.001). The area of positive HLA-A signal in areas associated with TUJ1 staining was significantly correlated with symptoms of pain (Pearson r = 0.69, *P* < 0.05, Figs. 6D–F).

## 4. Discussion

To gain new insight into the molecular landscape underlying neuropathic pain, the cellular composition and transcriptional landscape in human trigeminal nerves in health and injury were comprehensively characterized using single nuclei RNA sequencing, spatial transcriptomics, RNAscope, and immunohistochemistry. Comparison of the transcriptional landscape of human neuromas with and without pain led to the identification of several differentially expressed genes that might play a role in pain development and maintenance.

Among the most highly differentially expressed genes in painful and nonpainful neuromas, several are involved in immune and inflammatory responses. *HLA-A*, a member of the MHC class I antigens, and *NLRC5*, its trans-activator^52^ were highly upregulated in painful neuromas, and genetic variants in the HLA region have previously been associated with conditions including postherpetic neuralgia and traumatic-injury induced neuropathic pain.^63,84,99^ *CXCL2* and *CXCL8* were also upregulated in painful neuromas, confirming the growing body of literature that attributes chemokines a role in pain conditions affecting the trigeminal system^89^: in particular, CXCL2 (C-X-C motif ligand 2) expression is increased in the trigeminal ganglia of rats after infraorbital nerve constriction,^43^ whereas CXCL8 (C-X-C motif ligand 8) is increased in the serum of patients with migraine^27^ and trigeminal neuralgia.^57^ Other genes differentially expressed between painful and nonpainful samples include the basement membrane protein *NID2* expressed in nonmyelinating Schwann cells, the matrix metalloprotease *MMP19* expressed in profibrotic fibroblasts, and the riboflavin transporter *SLC25A3*, which warrant further investigation.

Single nuclei RNA sequencing highlighted the variety of cell types populating both healthy and injured human peripheral nerves. A high degree of heterogeneity was observed in fibroblasts, where subtypes of perineurial cells were selectively associated with healthy and injured nerves. Species differences were noted regarding fibroblast-expressed genes compared to previous reports: PI16, expressed in perineural cells in mice,^87^ was found in endoneurial cells; while NGFR, expressed in endoneurial fibroblasts in mice,^17,108^ was expressed in perineurial cells in our human samples. The neuromas were characterized by the presence of a profibrotic fibroblast cluster expressing *PRRX1*, which might contribute to scar formation and impair axonal regeneration.^6,55^ Both myelinating and nonmyelinating Schwann cells were identified, but the former exhibited greater heterogeneity. One subtype of myelinating Schwann cells (mSC_2) was marked by *PMP2* expression, previously identified as a marker for Schwann cells myelinating large-fiber neurons.^108^

We observed an expansion in the inflamed endothelial cell cluster in injured nerves compared to healthy trigeminal nerves, displaying an activated phenotype characterized by the expression of inflammatory markers such as *IL-6*, *SELE*, and *ICAM-1*. The endothelial cell cluster was more abundant in painful compared to nonpainful neuromas, displaying increased ligand–receptor interactions with Schwann and perineurial cells, involving MHC class II molecules, tight junctions, and FGF signaling. In addition, several of the differentially expressed genes identified by pseudo-bulk analysis are expressed in endothelial cells, including *NLRC5, JMJD1C, CXCL2, SLC25A3, ADGRG1*, and *TNFAIP3*. Endothelial cells might play a role in establishing a proinflammatory environment by enabling the influx of proinflammatory mediators and immune cells, which might contribute to pain chronification after nerve injury.

Previous work on human lingual neuromas investigated correlations between the expression of RNAs and proteins and symptoms of pain, focusing on ion channels including Na_v_1.7, 8, and 9, TRPA1, TRPV1, and the P2X7 receptor.^8–10,64,65,93,101^ Among the ion channels investigated, only Na_v_1.8 exhibited a correlation with symptoms of pain.^9^ On the other hand, the investigation of miRNA expression in neuromas identified several candidates, which are dysregulated in painful neuromas and putatively target interleukin and chemokine receptors.^93^

Other studies on human tissue have also identified inflammatory mediators to be linked with the presence of neuropathic pain. Ray et al.^76^ investigated the transcriptome of 50 human dorsal root ganglia derived from thoracic vertebrectomy, where a portion of the samples was associated with neuropathic pain. Despite detecting increased spontaneous activity in the DRG neurons associated with pain, the transcriptional changes detected were mainly linked with inflammation but not with ion channels expression or regulation.^70,76^ The most highly differentially expressed genes identified included Oncostatin M and *CXCL2* in males and genes linked with interferon signaling in females.^76^ In human Morton's neuromas, the presence of transcriptional signatures characteristic of macrophages and B cells was higher than in control samples and immunohistochemical analysis confirmed the increased density of CD163^+^ macrophages in neuromas compared to healthy nerve, whereas the density of CD68^+^ macrophages correlated with burning pain symptoms.^83^

The investigation of pathologically relevant human tissue is highlighting the prominent role that inflammation and the immune system play in neuropathic pain. Evidence from gene association studies identified the importance of several loci in the HLA region that contribute to neuropathic pain susceptibility or confer protection after peripheral nerve injury,^24^ complex regional pain syndrome,^20^ and diabetes,^62^ as well as in rat strains with different susceptibility to the development of mechanical allodynia after nerve injury.^25^ The functional effects of the variants identified have not been investigated; however, Escande-Beillard et al.^28^ have shown that neurons from retina explants respond to MHC class I allele products by inhibiting neurite outgrowth and reducing synaptic plasticity. Whether HLA has an effect on neuronal sensitization would be an interesting avenue for further investigation. One might hypothesize that specific variants in the HLA region might result in hyperactivity in the immune system where tissue injury triggers exaggerated immune responses that lead to maladaptive changes in the nervous system. This might occur via upregulation and increased antigen presentation, heightening immune activation, as well as by the potential direct activation of nerve fibers by HLA antigens.

A recent multiomic characterization of sural and tibial nerves from patients with moderate to severe diabetic peripheral neuropathy (DPN)^95^ described upregulation of CXCL2 in samples with DPN compared to controls, with expression in healthy nerves being higher in the sural component (containing only sensory fibers), compared to the tibial (also containing motor fibers). Alterations in cell-type composition were associated with severe axonal loss, including an increase in fibroblasts, immune cells, and endothelial populations. Overall, the results point to vascular remodeling and immune infiltration as critical features in the transition from moderate to severe axonal loss in DPN. A larger multiomic characterization of human samples with polyneuropathies of various etiologies^38^ enabled characterization of cell types at high resolution, identifying cell-type markers confirmed by our dataset, including *CXCL14* in perineurial cells and *PRIMA1* in nonmyelinating Schwann cells. A large number of differentially expressed genes were identified in endothelial cells comparing controls and polyneuropathies. We did not find the variety of immune cells reported by Heming et al.,^38^ possibly because of the different pathophysiologies in that study's cohort. Overall, there is increasing evidence linking the blood–nerve breakdown and immune infiltration to peripheral neuropathies. More large-scale analyses of multiple datasets are needed to establish the timeframe of cellular and molecular changes across different pathologies and dissect mechanisms linked with the transition from acute to chronic neuropathic pain.

CXCL2 and CXCL8 are both ligands for CXCR2. Several studies evidence CXCR2 upregulation in neurons of rat and mice DRG and spinal cord after nerve injury, whereas its inhibition is shown to attenuate neuronal hyperexcitability and behavioral responses to pain.^51,56,73,86,109,110^ The role of CXCL8-mediated activation of CXCR2 is particularly interesting for further investigation because of the evidence of CXCL8 upregulation in the serum of patients suffering from neuropathic pain conditions affecting the trigeminal system, such as migraines^27^ and trigeminal neuralgia.^57^

Overall, a picture is emerging where the immune system plays a significant role in whether an individual is likely to develop neuropathic pain after nerve injury. Further work to confirm the findings in this study on a larger number of samples is required, as well as functional investigation of the differentially expressed genes and whether they play a causative role in neuropathic pain development and maintenance and might act as targets for novel therapeutics, or if they can be detected systemically and used as biomarkers for patient stratification.

In summary, the work presented here provides a unique atlas describing the cell-type composition and transcriptional signatures in human healthy and injured trigeminal nerves, with and without neuropathic pain. The atlas highlights the role of endothelial cells in neuropathic pain, which display a proinflammatory phenotype and are more abundant in neuromas, particularly in the samples linked with pain, compared to healthy nerves. In addition, differential expression analysis indicated overexpression of *HLA-A* and *NLRC5*, involved in antigen presentation, and the chemokines *CXCL2* and *CXCL8*. Protein expression of HLA-A was localized to blood vessels and exhibited a correlation with symptoms of pain. These findings contribute to the idea that inflammation is a prominent aspect of neuropathic pain, with increased antigen presentation and chemokine signaling in painful neuromas. Further work is needed to identify whether genetic factors might predispose the immune system to a heightened response to injury and result in higher inflammation and pain chronification. Nevertheless, the atlas will represent a precious resource for the pain community to advance the understanding of neuropathic pain mechanisms, translate findings from animal models, and develop novel therapeutics.

## Conflict of interest statement

E.S. is a full-time employee of Eli Lilly and Company from which he receives benefits including stocks. T.J.P. is a cofounder of and holds equity in 4E Therapeutics, NuvoNuro, PARMedics, Nerveli, and Doloromics. T.J.P. has received research grants from AbbVie, Eli Lilly, Grunenthal, GSK, Evommune, Hoba Therapeutics, and The National Institutes of Health. D.W.L. has received research grants from the Medical Research Council (MRC), the Biotechnology and Biological Sciences Research Council (BBSRC), the Engineering and Physical Sciences Research Council (EPSRC), Yorkshire Cancer Research, Cancer Research UK, the Vivensa Foundation, Academy of Medical Sciences, the Royal Society, GSK, AstraZeneca, and Eli Lilly. He is a Trustee of the UK Society for Extracellular Vesicles. F.M.B. has received research grants from the Medical Research Council (MRC), the Biotechnology and Biological Sciences Research Council (BBSRC), the Engineering and Physical Sciences Research Council (EPSRC), Versus Arthritis, Eli Lilly, and Haleon. The remaining authors have no conflicts of interest to declare.

## Appendix A. Supplemental digital content

Supplemental digital content associated with this article can be found online at http://links.lww.com/PAIN/C443.

## Supplementary Material
