# Supplementary material for: Spatially resolved single-cell analysis of transcriptomic changes linked with neuropathic pain in human neuromas
Source: Pain. 2026 Jan 22;167(3):627–44. doi: 10.1097/j.pain.0000000000003907 (PMC12890192; doi:10.1097/j.pain.0000000000003907)
Supplement: Supplementary file 1 [file jop-167-627-s001.pdf]

Spatially resolved single-cell analysis of transcriptomic changes linked with neuropathic pain in human neuromas

Martina Morchio[a,†], Ishwarya Sankaranarayanan[b], Diana Tavares-Ferreira[b], Natalie Wong[a], Simon Atkins[a], Emanuele Sher[c], Theodore J. Price[b], Daniel W. Lambert[a], Fiona M. Boissonade[a,*]

[a]*Neuroscience Institute and School of Clinical Dentistry, University of Sheffield, Sheffield, UK;* [b]*Department of Neuroscience and Center for Advanced Pain Studies, University of Texas at Dallas, Richardson, TX, USA;* [c]*Eli Lilly and Company, Lilly UK Neuroscience Hub, Bracknell, UK*

**Corresponding author. Address: School of Clinical Dentistry, University of Sheffield, 19 Claremont Crescent, Sheffield, S10 2TA, UK. Tel.: +44 (0)114 215 9314.*
Email address: f.boissonade@sheffield.ac.uk

†*Current address: Centre for Regenerative Medicine, University of Edinburgh, Edinburgh, UK*

12 pages, 4 tables, 3 figures, 10 references

Table S1. Parameters and information for snRNAseq data analysis.

The table displays information on the snRNAseq dataset, including the metrics calculated by Cellranger in data preprocessing, the parameters used for Cellbender ambient RNA removal, the filtering parameters used in Seurat, as well as parameters used to perform integration and clustering.

	**N1**	**N2**	**TG1**	**TG2**
**Cellranger analysis**				
Cells detected	10,977	10,926	24,250	27,236
Confidently mapped reads in cells	75.14%	71.43%	74.36%	70.90%
Estimated UMIs from genomic DNA	1.14%	0.80%	0.04%	0.05%
Estimated UMIs from genomic DNA per unspliced probe	6	7	2	2
Median UMI counts per cell	1,536	2,781	2,893	2,582
Median genes per cell	983	1,550	1,773	1,666
Median reads per cell	5,321	9,519	10,754	9,887
Number of reads from cells called from this sample	79,134,506	134,083,140	474,861,720	426,518,737
Reads confidently mapped to filtered probe set	94.18%	95.52%	90.45%	87.99%
Reads confidently mapped to probe set	96.01%	97.21%	92.44%	89.74%
Reads mapped to probe set	99.25%	99.28%	99.23%	99.21%
Total genes detected	18,060	18,067	17,495	17,592
**Cellbender parameters and results**				
Expected cells	10000	10000	20000	20000
Total droplets included	50000	50000	70000	70000
fpr	0.01	0.01	0.01	0.01
learning-rate	0.00005	0.00005	0.00005	Default
epochs	150	150	200	200
counts in non-empty droplets removed	3.40%	3.10%	5.79%	8.68%

**Filtering parameters in Seurat**				
n Cells pre-filtering	15295.00	17392.00	25608.00	28536.00
nCount_RNA threshold	500.00			
nFeature_RNA threshold	250.00			
log10GenesPerUMI threshold	0.80			
percent.mt threshold	5.00			
nCells post-filtering	10892.00	11193.00	16053.00	18993.00
n clusters (res= 0.5)	19.00	19.00	14.00	16.00
**Integration and clustering in Seurat**				
normalization method	"SCT"			
reduction	"rpca"			
resolution	0.50			
communities	27.00			
n Cells after cleanup of clusters	10847	11143	16047	18922

Table S2. Summary of the marker genes used for cell type annotation.

Markers derived from the literature [1-3,5,7,8] used to annotate each cell type are listed.

Cell type	Marker genes
General fibroblasts	DCN, GSN, VIM, COL1A1, FN1
Endoneurial fibroblasts	OSR2, ABCA8, ABCA9, ABCA10, PLXDC1, COL15A1
Perineurial fibroblasts	CLDN1, SLC2A1, PTCH1, LMO7, ITGB4, KLF5, NGFR
Meningeal fibroblasts	OGN, PTGDS, FXYD5, ALPL, CRABP2
Endothelial cells	EGFL7, PECAM1, TIE1, EMCN, CDH5, VWF, CLDN5, ECSCR
Vascular smooth muscle cells / pericytes	TPM2, MYH11, ACTA2, MYLK, PDGFRB
Schwann cells	SOX10, PLP1, ERBB3, NCAM1, S100B
Non-myelinating Schwann cells	L1CAM, NRXN1, NCAM1
Myelinating Schwann cells	MBP, MPZ, EGR2, NCMAP
Damaged Schwann cells	ATF3, EGR1, FOS, JUN
Repair Schwann cells	NGFR, BDNF, GDNF, ERBB3, SOX2, CADM1, ATF3, RUNX2
Lymphocytes	PTPRC, CD3G, CXCR6, TRAC, CD3E, SKAP1, THEMIS, IL7R
Myeloid	AIF1, CD68, MRC1, SIGLEC1, ITGAM, CSF1R
Myocytes	MYL1, TNNT1, TNNT3, TNNI1
Oligodendrocytes	OLIG2, OLIG1, MOG, CNP, PLP1
Astrocytes	GFAP
Salivary gland cells	MUC5B, AQP5, KRT19, KRT7, KRT14

Table S3. Differential abundance analysis

Differential abundance of cell types between painful and non-painful samples analyzed with spatial transcriptomics was calculated with EgdeR using the quasi-likelihood negative binomial generalized log-linear model. For each cell type, the log fold change of relative abundance, the p value and the false discovery rate (FDR) calculated with the Benjamini-Hochberg method are shown.

	**logFC**	**P-value**	**FDR**
SC6	3.04	0.02	0.17
SC2	2.47	0.02	0.17
SC3	1.26	0.03	0.17
Endo	0.80	0.09	0.33
SC4	1.08	0.11	0.34
Myo1	-3.09	0.15	0.38
SC5	-1.19	0.23	0.40
Myo2	-2.40	0.23	0.40
Myo4	-2.47	0.24	0.40
Macro	1.43	0.28	0.42
Myo3	-1.83	0.31	0.43
Peri	0.52	0.40	0.48
Fibro	0.37	0.42	0.48
SC1	-0.45	0.45	0.48
Bcells	0.48	0.66	0.66

Table S4. Top five marker genes for each cluster and the putative enriched cell-type.

Each cluster was annotated based on the top differentially expressed genes, where a putative cell type enriched in each barcode was assigned based on gene expression. The number of spots classified as each cell type across all samples is also reported.

Cluster number	Cluster name	Top five DE genes					N of spots	Putative enriched cell type
1	Fibro	COL1A1	SFRP2	FBLN1	COL1A2	SFRP4	4607	Fibroblast
2	Endo	AQP1	CCL14	IL6	TM4SF1	SELE	4422	Endothelial cells
3	SC1	HBA2	MPZ	HBA1	PMP22	MBP	4327	Schwann cells
4	Myo1	MB	TNNT1	TCAP	CKM	TTN	4214	Myocytes
5	Peri	PTGDS	CLDN1	SLC2A1	IGFBP6	MPZ	3873	Perineurial cells
6	SC2	MBP	PMP22	MPZ	PRX	S100B	3571	Schwann cells
7	Myo2	MB	TNNT1	TCAP	CKM	TNNI1	2936	Myocytes
8	SC3	PMP22	MBP	MPZ	PRX	APOD	2882	Schwann cells
9	Myo3	MB	TNNT1	CKM	TCAP	TTN	2458	Myocytes
10	SC4	APOD	MPZ	PMP22	MBP	PRX	2162	Schwann cells
11	Myo4	ACTC1	THBS4	MYLPF	CA3	COL1A1	1937	Myocytes
12	SC5	MPZ	PRX	MBP	PMP22	S100B	1549	Schwann cells
13	Bcells	IGKC	IGHG2	APOD	IGHG1	S100B	1083	B cells
14	SC6	PMP22	MBP	MPZ	PRX	S100B	449	Schwann cells
15	Macro	LYZ	MMP9	SPP1	LAPTM5	CHIT1	353	Macrophages
16	NA	PRX	APOD	MPZ	CNP	MBP	8	NA
17	NA	GPM6B	EGR2	MPZ	CARD8	FGFBP2	5	NA

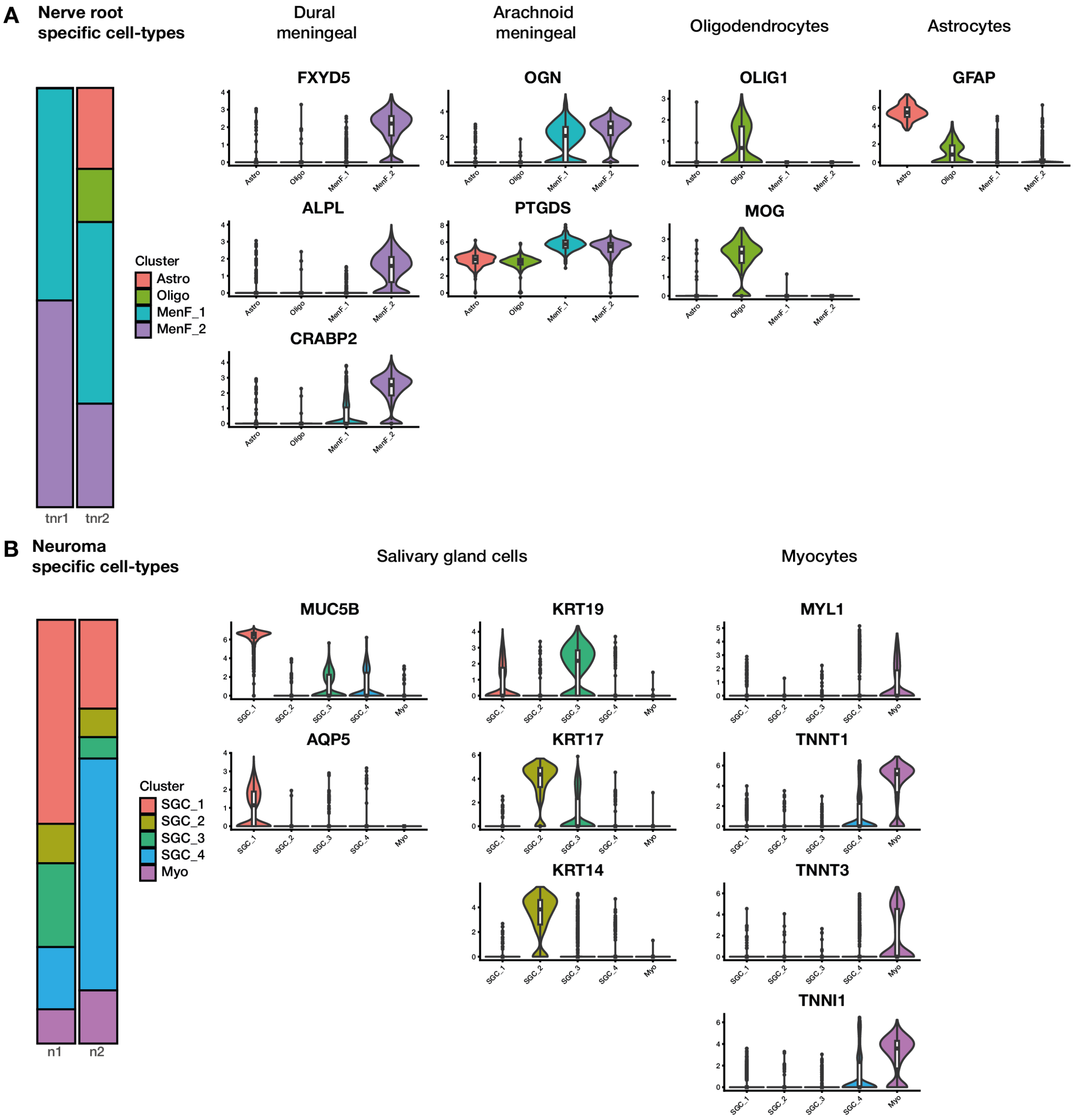

Figure S1. Sample-specific cell-types identified by snRNA-seq.

Nerve root (**A**) and neuroma (**B**) specific cell types identified by snRNA seq. In nerve root samples, meningeal fibroblasts are identified by arachnoid markers (MenF_1: OGN, PTGDS) and dural markers (MenF_2: FXYD5, ALPL, CRABP2)[3]. Astrocytes are identified by GFAP expression, while oligodendrocytes by OLIG1 and MOG expression [10]. In the neuroma samples, salivary gland cells (SGC) are identified by the MUC5B and AQP5 expression, typically expressed by acinar cells (SGC_1), KRT14 and 17, expressed by basal duct cells (SGC_2) and KRT19 expressed by ductal cells (SGC_3) [4,6]. Myocytes are identified by the expression of troponin genes [9].

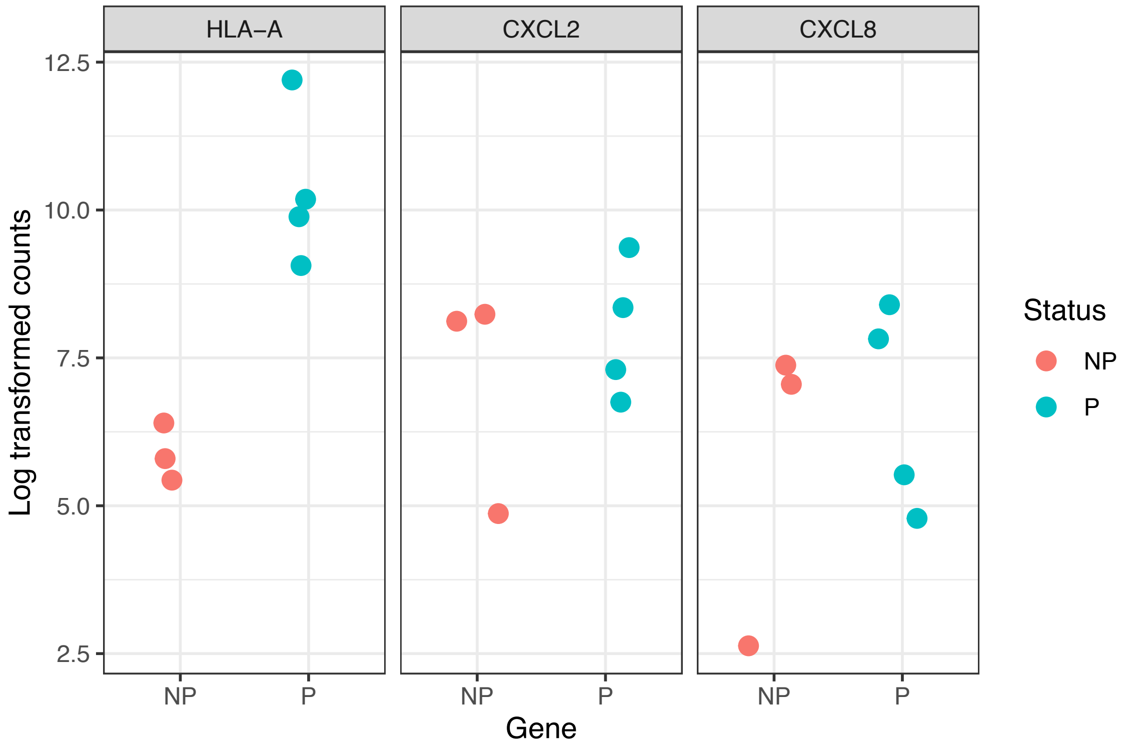

Figure S2. Log-normalized counts for HLA-A, CXCL2 and CXCL8 from pseudo-bulk spatial transcriptomics in painful and non-painful samples.

NP, non-painful; P, painful.

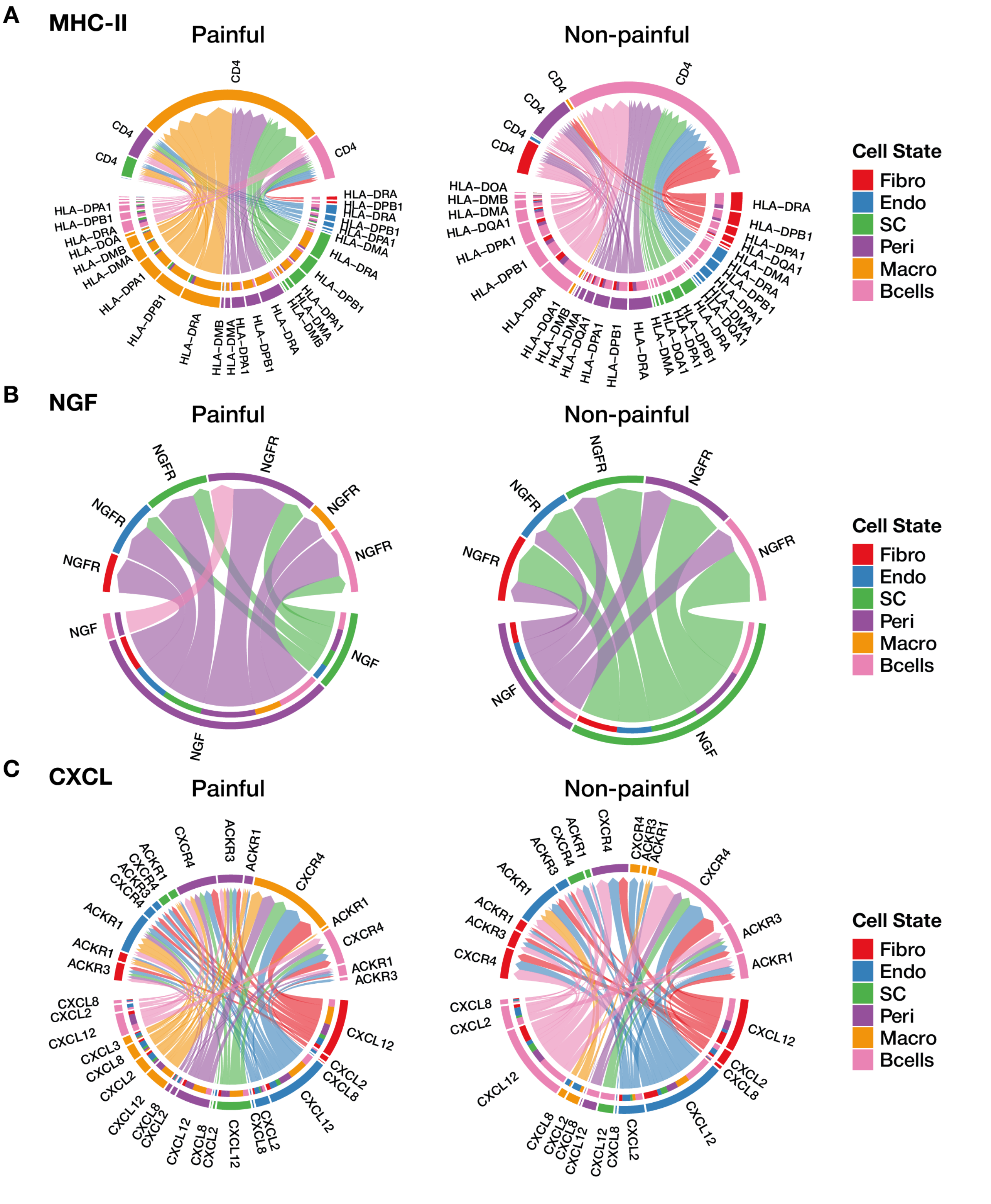

Figure S3. Ligand-receptor interactions in painful and non-painful samples in the MHC-II, NGF and CXCL pathways.

Chord plots displaying the inferred ligand-receptor interactions in painful (left) and non-painful (right) samples for the MHC-II (**A**), NGF (**B**) and CXCL (**C**) pathways. The chords are color coded by the sender cell-type and directed towards the receiver, whose cell-type is color coded in the targeted segment. The ligands expressed by the senders are displayed at the bottom, while the receptors expressed by the receivers are displayed at the top.

References

[1] Chau MJ, Quintero JE, Monje PV, Voss SR, Welleford AS, Gerhardt GA, van Horne CG. Using a transection paradigm to enhance the repair mechanisms of an investigational human cell therapy. Cell Transplant 2022;31:9636897221123515. https://doi.org/10.1177/09636897221123515.

[2] Chen B, Banton MC, Singh L, Parkinson DB, Dun XP. Single cell transcriptome data analysis defines the heterogeneity of peripheral nerve cells in homeostasis and regeneration. Front Cell Neurosci 2021;15:624826. https://doi.org/10.3389/fncel.2021.624826.

[3] DeSisto J, O'Rourke R, Jones HE, Pawlikowski B, Malek AD, Bonney S, Guimiot F, Jones KL, Siegenthaler JA. Single-cell transcriptomic analyses of the developing meninges reveal meningeal fibroblast diversity and function. Dev Cell 2020;54(1):43-59. https://doi.org/10.1016/j.devcel.2020.06.009.

[4] Faruque M, Wanschers M, Ligtenberg AJ, Laine ML, Bikker FJ. A review on the role of salivary MUC5B in oral health. J Oral Biosci 2022;64(4):392-399. https://doi.org/10.1016/j.job.2022.09.005.

[5] Gong T, Wang Y, Dong S, Ma X, Du D, Zou C, Zheng Q, Wen Z. Single-cell RNA-seq reveals the communications between extracellular matrix-related components and Schwann cells contributing to the earlobe keloid formation. Front Med (Lausanne) 2022;9:1000324. https://doi.org/10.3389/fmed.2022.1000324.

[6] Hauser BR, Aure MH, Kelly MC, Hoffman MP, Chibly AM. Generation of a single-cell RNAseq atlas of murine salivary gland development. iScience 2020;23(12):101838. https://doi.org/10.1016/j.isci.2020.101838.

[7] Heming M, Börsch A-L, Wolbert J, Thomas C, Mausberg AK, Szepanowski F, Eggert B, Lu IN, Tietz J, Dienhart F, Meschnark M, Strecker J-K, Glatza M, Thomas C, Gmahl N, Dambietz C, Müther M, Uerschels A-K, Keyvani K, Minnerup J, Doppler K, Üçeyler N, Aprea J, Dahl A, Stassart R, Fledrich R, Wiendl H, Sommer C, Stettner M, Meyer zu Hörste G. Multi-omic identification of perineurial hyperplasia and lipid-associated nerve macrophages in human polyneuropathies. Nat Commun 2025;16(1):7872. https://doi.org/10.1038/s41467-025-62964-8.

[8] Lovatt D, Tamburino A, Krasowska-Zoladek A, Sanoja R, Li L, Peterson V, Wang X, Uslaner J. scRNA-seq generates a molecular map of emerging cell subtypes after sciatic nerve injury in rats. Commun Biol 2022;5(1):1105. https://doi.org/10.1038/s42003-022-03970-0.

[9] Rasmussen M, Jin J-P. Troponin variants as markers of skeletal muscle health and diseases. Front Physiol 2021;12:747214. https://doi.org/10.3389/fphys.2021.747214.

[10] Scolding NJ, Frith S, Linington C, Morgan BP, Campbell AK, Compston DA. Myelin-oligodendrocyte glycoprotein (MOG) is a surface marker of oligodendrocyte maturation. J Neuroimmunol 1989;22(3):169-176. https://doi.org/10.1016/0165-5728(89)90014-3.
